# Structure and Catalytic Mechanism of a Bacterial Friedel–Crafts Acylase

**DOI:** 10.1002/cbic.201800462

**Published:** 2018-11-26

**Authors:** Tea Pavkov‐Keller, Nina G. Schmidt, Anna Żądło‐Dobrowolska, Wolfgang Kroutil, Karl Gruber

**Affiliations:** ^1^ Austrian Centre of Industrial Biotechnology (ACIB) Petersgasse 14 8010 Graz Austria; ^2^ Institute of Molecular Biosciences University of Graz Humboldtstrasse 50 8010 Graz Austria; ^3^ Department of Chemistry, Organic and Bioorganic Chemistry University of Graz Heinrichstrasse 28/2 8010 Graz Austria; ^4^ BioTechMed-Graz Mozartgasse 12/II 8010 Graz Austria

**Keywords:** acyltransferases, Friedel–Crafts acylation, multicomponent enzymes, solid-state structures, transferases, X-ray diffraction

## Abstract

C−C bond‐forming reactions are key transformations for setting up the carbon frameworks of organic compounds. In this context, Friedel–Crafts acylation is commonly used for the synthesis of aryl ketones, which are common motifs in many fine chemicals and natural products. A bacterial multicomponent acyltransferase from *Pseudomonas protegens* (*Pp*ATase) catalyzes such Friedel–Crafts C‐acylation of phenolic substrates in aqueous solution, reaching up to >99 % conversion without the need for CoA‐activated reagents. We determined X‐ray crystal structures of the native and ligand‐bound complexes. This multimeric enzyme consists of three subunits: PhlA, PhlB, and PhlC, arranged in a Phl(A_2_C_2_)_2_B_4_ composition. The structure of a reaction intermediate obtained from crystals soaked with the natural substrate 1‐(2,4,6‐trihydroxyphenyl)ethanone together with site‐directed mutagenesis studies revealed that only residues from the PhlC subunits are involved in the acyl transfer reaction, with Cys88 very likely playing a significant role during catalysis. These structural and mechanistic insights form the basis of further enzyme engineering efforts directed towards enhancing the substrate scope of this enzyme.

## Introduction

Acyltransferases belong to the EC 2.3 groups of enzymes. This subclass contains enzymes that transfer an acyl group from a donor compound to the hydroxy, amino, or mercapto group of an acceptor compound, forming an ester, amide, or thioester. Different acyltransferase classes have been identified in living organisms that utilize, for instance, 1‐*O*‐acylglucosides,^1^ acylated acyl carrier proteins,^2^ or quinic acid ester^3^ as high‐energy activated acyl donors. The most abundant group of acyltransferases mostly require acyl‐CoA derivatives as donor substrates, catalyzing various reactions involved in primary and secondary metabolism.^4^ In bacteria, CoA‐dependent O‐ and N‐acylation play a key role in detoxification of antibiotics such as chloramphenicol,^5^ aminoglycosides,^6^ streptothricin,^7^ and phosphinothricin.^8^ Acyltransferases are also involved in numerous biosynthetic pathways, especially in the biosynthesis of membrane phospholipids,^9^ of wax esters and triacylglycerols,^10^ and of polyketides,^11^ and also play a role in lysozyme resistance.^12^

In contrast to O‐ and N‐acylation, natural C‐acylation reactions are scarce. So far, only a couple of acyltransferases, for example, from *Pseudomonas protegens* (*Pp*ATase) and *Pseudomonas fluorescens* have been reported to catalyze C−C bond formation, in the biosynthesis of the antibiotically active polyketide 1,1′‐(2,4,6‐trihydroxybenzene‐1,3‐diyl)bisethanone (diacetylphloroglucinol, DAPG).^13^ The biosynthesis of DAPG is regulated by the phlACBDEFGHI gene cluster, which is divided into regulatory genes phlEFGHI and the biosynthetic operon phlACBD.^14^ For many years, biocatalytic applications were limited to gene phlD, which encodes a type‐III polyketide synthase that was employed for the in vivo production of the DAPG precursor benzene‐1,3,5‐triol (phloroglucinol, PG) either in *Pseudomonas* sp. or in *Escherichia coli* under controlled conditions in bioreactors.13b, 15

**Scheme 1:**
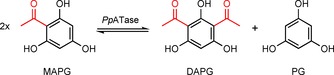
Natural reaction catalyzed by the *P. protegens* acyltransferase (*Pp*ATase) involved in the biosynthesis of DAPG.

Very recently, the scope of applications has been extended to phlACB, which encodes the cofactor‐independent acyltransferase *Pp*ATase referred to above. This catalyzes the reversible disproportionation of 1‐(2,4,6‐trihydroxyphenyl)ethanone (monoacetylphloroglucinol, MAPG) into PG and DAPG in a divergent reaction (Scheme 1). *Pp*ATase is a multicomponent enzyme and is catalytically active without addition of cofactors such as CoA or ATP. A functional enzyme is only obtained upon expression of the entire phlABC operon: mixing and incubation of the individually expressed proteins—PhlA, PhlB, and PhlC—with MAPG did not lead to its disproportionation.13a, 16

It was shown that *Pp*ATase accepts various C‐ or O‐acyl donors such as isopropenyl acetate and transfers an acetyl moiety to a phenolic acceptor in a Friedel–Crafts‐type acetylation.16b, 17 The enzyme complex also shows chemical reaction promiscuity and accepts aniline derivatives as substrates for amide formation.^18^

Here we report on the crystal structure determination of *Pp*ATase in its substrate‐free form, as well as after soaking of the crystals with MAPG. The structural results indicate that only one of the three subunits—PhlC—is involved in catalyzing the acylation reaction. Site‐directed mutagenesis studies and activity measurements complement the structural data and enable a catalytic mechanism to be proposed.

## Results and Discussion

### Overall structure

We determined X‐ray crystal structures of a CoA‐independent acyltransferase produced by *P. protegens* DSM 19095 (*Pp*ATase) using diffraction data from two different crystal forms at 2.8 and 3.4 Å resolution, respectively (Table 2 in the Experimental Section and Figure S1 in the Supporting Information). The structures were solved by molecular replacement involving extensive density modification as well as automated and manual rebuilding (see the Experimental Section). It had been known before that *Pp*ATase is a multimeric enzyme consisting of three subunits—PhlA, PhlB, and PhlC.^19^ The final structures showed the hexagonal crystal form (space group *P*6_1_22) to contain two copies of each of those subunits in the asymmetric unit and the orthorhombic crystal form (space group *P*2_1_2_1_2_1_) to contain eight copies of each subunit.

**Figure 1:**
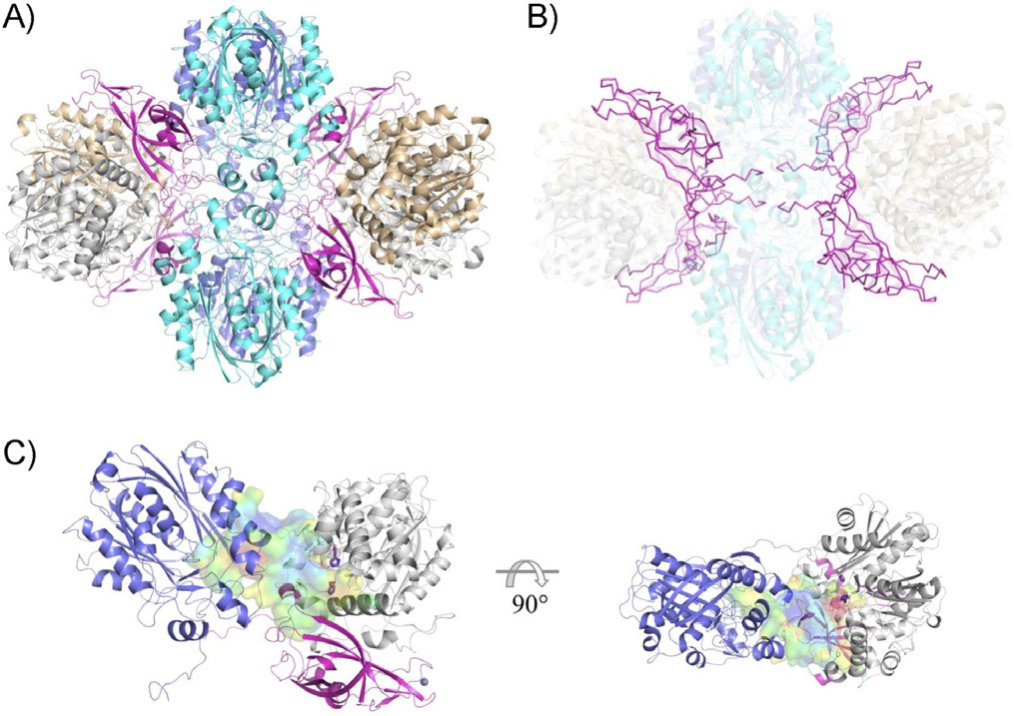
Structural analysis of *Pp*ATase. A) Structure of the heterododecamer Phl(A_2_C_2_)_2_B_4_ (PhlA=blue and cyan, PhlB=magenta, PhlC=gray and light brown). B) PhlB mediates the binding of two PhlA and two PhlC dimers. Two N‐terminal tails of PhlB (up to Arg28) are involved in tight interactions with adjacent PhlA and PhlC molecules as well as with the N‐terminal tail of a neighboring PhlB molecule. PhlB is shown in ribbon representation. C) The continuous cavity between PhlA, PhlB, and PhlC subunit trimer. The active site of PhlC (in violet, residues shown in stick representation) is situated adjacent to the cavity of PhlA. The long loop in PhlB (Glu74–Val87, magenta) is closing the cavity from the other side. The cavity is shown in surface representation (red/hydrophobic to blue/hydrophilic). See the Experimental Section for detailed descriptions.

In both structures, an analysis of protein–protein interactions within the crystal by using the EBI‐Pisa server^20^ yielded a heterododecamer with four copies of each subunit as the most likely biologically active oligomer of *Pp*ATase. Thus, the hexagonal crystal form contains half a dodecamer in the asymmetric unit with the other half being generated by a crystallographic diad. The orthogonal crystals, on the other hand, contain two copies of the full dodecamer in the asymmetric unit. Closer inspection of the inter‐chain contacts indicated that PhlA and PhlC each form strongly interacting homodimers in the crystal (Figure S2). The composition of the multimeric enzyme complex is thus best described as Phl(A_2_C_2_)_2_B_4_, in which the four copies of PhlB mediate the binding of two PhlA and two PhlC dimers (Figure 1 B) to form the complete oligomer (Figure 1 A and B).

A cavity analysis identified four, large, contiguous cavities per dodecamer, lined by residues from one particular copy of PhlA, PhlB, and PhlC. In this heterotrimeric arrangement the proposed active sites of PhlA and PhlC (see below) are adjacent to each other on one side of the cavity, whereas PhlB (especially through a long loop formed by residues Glu74 to Val87) closes the cavity on the opposite side (Figure 1 C).

### Comparison with other structures

We used the Dali server,^21^ as well as PDBeFold,^22^ to identify structurally similar proteins in the PDB. According to these analyses, the closest structural homologues to PhlA are hydroxymethylglutaryl‐CoA synthases (e.g., the enzyme MvaS from *Myxococcus xanthus*, PDB ID: 5HWQ)^23^ and two β‐oxoacyl‐(acyl‐carrier‐protein) synthases: from *Aquifex aeolicus* (PDB ID: 2EBD, RIKEN Structural Genomics/Proteomics Initiative) and from *E. coli* (PDB ID: 1HNH).^24^ The sequence identity of PhlA with these homologues is below 24 % with root‐mean‐square‐deviations (rmsds) of approximately 2 Å.

Previous sequence analyses had suggested that PhlA could be involved in the first step of DAPG biosynthesis: that is, the formation of acetoacetyl‐CoA from acetyl‐CoA.^25^ Inspections of the cavity observed in our structures in the vicinity of PhlA suggests that there is indeed enough space for acetyl‐CoA binding. However, key residues necessary to catalyze this Claisen‐type condensation (a cysteine and a glutamic acid residue) are not present in PhlA. Comparison with homologous enzymes shows the crucial cysteine residue corresponding to Gly115 in PhlA and the active‐site glutamate residue aligning with Cys83 in PhlA. We attempted to obtain a complex structure by soaking *Pp*ATase with acetyl‐CoA, but the soaked crystal did not diffract sufficiently well for structure determination.

Structural analysis of PhlB suggested a close similarity to a protein of unknown function from *Sulfolobus solfataricus* (PDB ID: 3IRB, rmsd 2.6 Å, seq‐id: 18 %).^26^ This protein from the DUF35 family (Pfam PF01796) exhibits a two‐domain architecture consisting of an N‐terminal, rubredoxin‐like zinc ribbon and a C‐terminal oligonucleotide/oligosaccharide‐binding (OB) fold domain^27^ with an additional N‐terminal helical segment possibly involved in protein–protein interactions^26^ (Figure S3). For members of this protein family, a general role in fatty acid and polyketide biosynthesis as acyl‐CoA‐binding proteins has been predicted.^26^

Although a similar domain organization is observed in PhlB, and the zinc ribbon with its Cys_4_ metal‐ion‐binding site is conserved, severe differences are evident in the OB domain and at the N terminus. Instead of two N‐terminal helices, PhlB exhibits an elongated N‐terminal tail and a short, kinked α‐helix. In the dodecameric arrangement of *Pp*ATase, this region of PhlB (up to Arg28) is not accessible to the solvent but buried within the enzyme complex structure, where it is involved in tight interactions with the adjacent PhlA and PhlC molecules, as well as with the N‐terminal tail of a neighboring PhlB molecule (Figure 1 B). The same is true for the two long loop regions within the OB domain (residues 73 to 89 and 127 to 136).

Finally, the sequence of PhlC shows that it belongs to the thiolase superfamily,^28^ although sequence identities to members of this enzyme family are below 45 %. The structure of this subunit exhibits an α/β‐hydrolase‐type fold and is most similar to structures of the thiolase‐like protein ST0096 from *Sulfolobus tokodaii* (PDB ID: 4YZO, rmsd 1.6 Å, seq‐id: 26 %) and the SCP2 thiolase from *Trypanosoma brucei* (PDB ID: 4BI9, rmsd 1.7 Å, seq‐id: 24 %).^29^ In thiolases, a cysteine and a histidine residue (corresponding to Cys88 and His347 in PhlC) are highly conserved and were found to be important for the enzymatic reaction. Beside those two residues, the active‐site cavity of PhlC is lined by amino acid residues His56, Asn87, His144, Trp211, Tyr298, and Ser349, which might play a role in substrate binding or catalysis (Figure S7). Even in the untreated (hexagonal) crystal, residual density was observed at the side chain of Cys88, thus indicating that this residue is at least partially acetylated.

Recently, the structure of an archaeal acetoacetyl‐CoA thiolase/HMG‐CoA synthase (HMGCS) complex from *Methanothermococcus thermolithotrophicus* was reported.^30^ The arrangement of the individual protein subunits within this complex is very similar to that in *Pp*ATase: the HMGCS subunit corresponds to PhlA in *Pp*ATase (sequence identity 38.5 %), whereas the thiolase subunit corresponds to PhlC (sequence identity 28.9 %). The complex also contains a protein from the DUF35 family that is related to PhlB in *Pp*ATase (sequence identity 26.7 %). Sequence alignments of the three separate subunits are shown in Figures S4–S6. In contrast to the *PpATase*, however, this complex utilizes acetyl‐CoA as an acyl donor and is involved in the mevalonate pathway. The HMGCS subunit is involved in the exergonic condensation of acetoacetyl‐CoA and acetyl‐CoA to form 3‐hydroxy‐3‐methylglutaryl‐CoA. The key Cys and Glu residues necessary to catalyze this Claisen‐type condensation are indeed present in HMGCS (Cys114 and Glu82), whereas they are missing in PhlA as mentioned above (Figure S8). The presence of the active Cys residue in the thiolase subunit is preserved in both complexes (Cys88 for PhlC and Cys85 in the thiolase subunit). Soaking of the thiolase/HMGCS complex with acetyl‐CoA (PDB ID: 6ESQ) revealed the binding of CoA at the subunit interface comprised by residues from all three proteins.^30^ This region is significantly different from its counterpart in the structure of *Pp*ATase (Figure S9), showing altered relative positions of individual secondary structure elements, the presence of additional residues in the PhlB region of *Pp*ATase, and two additional short β‐strands in HMGCS, as well as a lack of CoA‐binding residues in *Pp*ATase. The overall fold of PhlB is very similar to that in the protein from the DUF35 family in the thiolase/HMGCS complex (Figure S3). The elongated N terminus of PhlB that is buried within the *Pp*ATase complex structure, however, is completely missing in the other structure, and notable conformational changes are observed in the opposite loop region.

### Structure of the complex with MAPG

**Figure 2:**
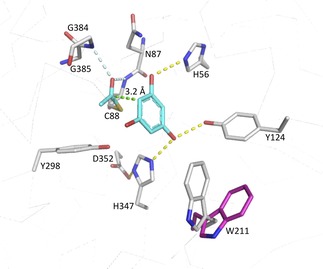
Active‐site structure of PhlC after soaking with MAPG. This depicts the intermediate formed upon acetyl transfer from the substrate to Cys88 of the enzyme (the transforming co‐product PG and the transferred acetyl group are depicted in cyan). Trp211 can act as a lid covering the entrance to the active site (closed in gray, open in magenta). Hydrogen bonds are shown as yellow dashed lines; the contact between the carbonyl carbon atom of the acetyl group and C‐6 of the substrate aromatic ring is indicated as a green dashed line. The carbonyl oxygen atom of the Cys88‐bound acetyl group is positioned in the “oxyanion hole” of the α/β‐hydrolase‐type fold, forming hydrogen bonds (indicated by the light blue dashed lines) with the main‐chain amide groups.

In order to identify the subunit responsible for the observed transferase activity, we performed crystal‐soaking experiments. Orthorhombic crystals of *Pp*ATase were soaked with the native acetyl donor/acceptor MAPG, and the structure of the complex was solved at 3.4 Å resolution. Residual density was observed in the vicinity of residue Cys88 in four out of the eight crystallographically independent copies of the PhlC subunit (Figure S10). This density was compatible with an acetylated Cys88 residue and with a (deacetylated) PG molecule bound at this site (Figure 2). The resulting structure revealed that only residues from PhlC interact with the bound PG. Specifically, the side chains of His56 and of Tyr124 and His347 form hydrogen bonds with the hydroxy groups at positions 1 and 3, respectively, of the aromatic ring (Figure 2). In contrast, the hydroxy group at position 5 is apparently not involved in any direct interactions with PhlC. Instead, it points towards a small cavity lined by mostly hydrophobic residues (such as Phe148, Leu209, and Leu300).

The carbonyl oxygen atom of the Cys88‐bound acetyl group is positioned in the “oxyanion hole” of the α/β‐hydrolase‐type fold forming hydrogen bonds with the main‐chain amide groups of Cys88 itself and of Gly385. Additional polar stabilization might be provided by the helix dipole oriented favorably towards Cys88 (the “nucleophile elbow” present in this type of fold^31^). The acetyl group is oriented more or less parallel to the aromatic ring, and its carbonyl carbon atom is appropriately positioned for an electrophilic attack on the C‐6 atom of the substrate, with distances between the two atoms ranging from 3 to 3.5 Å (Figure 2). Its methyl group points towards Phe148.

Whereas the conformations of most residues forming the active site of PhlC are similar in the structures, Trp211 in particular stands out because it adopts an open conformation in the untreated crystal and closes the cavity upon substrate binding in the soaked crystal (Figure 2).

### Mutagenesis of PhlC active‐site residues

The analysis of the PhlC active site identified Cys88, His144, Asn87, His56, Ser349, Tyr124, Tyr298, Asp352, His347, and Trp211 as a group of mostly polar or charged residues lining the cavity (Figure S7). Those residues were selected for site‐directed mutagenesis, and we analyzed the activity of the corresponding enzyme variants in the acetylation of resorcinol (**1**) with use of isopropenyl acetate (IPEA) as acetyl donor and imidazole (see the Experimental Section). The results are shown in Table 1 in the form of relative amounts of the reactant **1**, the C‐acetylation product 4‐acetylresorcinol (**2**), and the O‐acetylation product 2‐(3‐hydroxyphenyl)acetate (**3**).

**Table 1:** Activity of *Pp*ATase variants for the Friedel–Crafts acetylation of benzene‐1,3‐diol (resorcinol, **1**) and specific activity for the natural reaction.

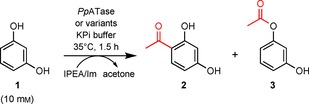					
	*Pp*ATase	Acetylation with IPEA/Im^[a]^			Specific activity
	variant	**1** [%]	**2** [%]	**3** [%]	[mU mg^−1^]^[b]^
1	w/o enzyme	68	n.d.	32	n.d.
2	wt enzyme	41	44	15	44
3	H56A	81	n.d.	19	n.d.
4	H56S	81	n.d.	19	n.d.
5	N87A	84	6	10	4
6	C88A	74	n.d.	26	n.d.
7	C88S	69	n.d.	31	n.d.
8	H144A	79	5	16	n.d.
9	H144S	81	n.d.	19	n.d.
10	W211A	31	66	3	16
11	W211F	48	40	12	24
12	Y298A	79	3	18	1
13	Y298V	82	n.d.	18	n.d.
14	Y298F	82	3	15	5
15	H347F	77	1	22	<1
16	S349A	73	n.d.	27	n.d.
17	D352V	73	1	26	<1

[a] Conditions: cell‐free extract of *Pp*ATase or variant (30 mg protein), benzene‐1,3‐diol (resorcinol; **1**, 10 mm), potassium phosphate buffer (50 mm, pH 7.5, total volume 1 mL), IPEA (100 mm), and imidazole (100 mm added from a 1 m stock solution prepared in the reaction buffer), 35 °C, 1.5 h, 750 rpm. The relative amounts of **1**–**3** were determined by HPLC according to standard curves with authentic samples. n.d.: not detected.^[b]^ Specific activity was determined spectrophotometrically by monitoring the disproportionation of MAPG into DAPG and PG.

As already shown previously,16b the reaction performed in the presence of the wild‐type enzyme produces predominantly the C‐acetylation product **2** (entry 2), whereas the non‐enzymatic reaction (entry 1) yields only the O‐acetylation product **3** under these conditions. Replacing Cys88 by alanine or serine completely abolishes ATase activity (entries 6 and 7). The same is true for replacements of His56 (entries 3 and 4), whereas exchanges of Asn87 (entry 5), His144 (entries 8 and 9), Tyr298 (entries 12–14), His347 (entry 15), Ser349 (entry 16), and Asp352 (entry 17) yielded variants that retained minute activities in some cases. As anticipated, the exchange of Trp211 for alanine or phenylalanine did not abolish the enzymatic activity of *Pp*ATase (entries 10 and 11). Use of the variant W211A, however, significantly shifted the product spectrum in favor of the C‐acetylation product.

### Proposed mechanism of acyl transfer

*Pp*ATase catalyzes the reversible acetylation of MAPG into PG and DAPG.^13^ It has been shown that only a multicomponent complex consisting of PhlA, PhlB, and PhlC subunits catalyzes the disproportionation of MAPG.13a, 16 The crystal structures presented here revealed that only residues from PhlC interact with the bound PG, thus strongly suggesting that PhlA and PhlB are not directly involved in the actual acyl transfer step. Instead, these subunits are very likely required for the formation of a properly folded and functional dodecameric enzyme complex. This finding also supports previous suggestions relating to their possible involvement in preceding steps of DAPG biosynthesis.^25^

**Scheme 2:**
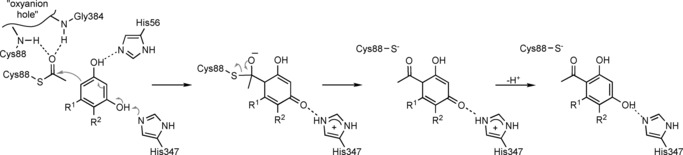
Proposed mechanism of the acyl transfer.

Combining the structural results with activity data obtained for enzyme variants (Table 1) allows a plausible catalytic mechanism to be proposed (Scheme 2). The observation of an acetylated Cys88 residue in both crystal structures and the lack of activity measured for the cysteine‐to‐alanine variant indicates that Cys88 very likely plays a significant role during catalysis. We suggest that the thiol group of Cys88 attacks the acyl moiety of the donor and subsequently forms a covalent intermediate similar to the acyl‐enzyme intermediates formed by serine or cysteine hydrolases.^32^ As in these hydrolases, the nucleophilic attack is facilitated by stabilization of the ensuing negative charge at the acyl oxygen atom by polar interactions within the “oxyanion hole”. There is no clear indication of a base, which could activate the thiol by deprotonation, in the vicinity of Cys88. It is reasonable, however, that the side chain already be deprotonated to some extent at neutral or slightly basic pH values. Additional stabilization of the thiolate is possible through polar interactions with the OH group of Tyr298 and the imidazole moiety of His347, although the distances to both groups are longer (>3.6 Å) than observed for typical hydrogen bonds. The proposed formation of an acyl‐enzyme intermediate is also consistent with our previous finding that conversions with DAPG/MAPG as acetyl donor did not yield any phenyl acetate derivatives and that the enzyme also catalyzes an intermolecular Fries rearrangement.16b

The second step of the reaction involves the transfer of the acyl moiety from Cys88 to the aromatic ring of an acceptor molecule. As discussed above, the carbonyl carbon of the cysteine‐bound acetyl group is appropriately positioned to attack the C‐6 atom of the bound PG in the complex structure (Scheme 2). Electronic activation of the aromatic ring most likely involves deprotonation of the phenolic OH group(s) at C‐1 (by His56) and/or at C‐3 (by the diad His347–Asp352). We have previously shown that, in addition to PG, resorcinol—but not phenol—can act as an acyl acceptor,16b thus indicating that at least two OH groups (at positions 1 and 3) are necessary for substrate binding and/or activity. In the structure, the OH group at C‐5 of PG does not participate in any polar interactions with the enzyme and it can indeed be replaced by other (alkyl) substituents in *Pp*ATase substrates.16b The rearomatization step through an intramolecular proton transfer very likely does not involve any enzyme intervention.

PhlC of *Pp*ATase differs from other, more common thiolases by the presence of the tryptophan residue Trp211. This residue appears to have a lid function, because its conformation significantly changes upon binding of the substrate (Scheme 2). This conformational flexibility could very well influence the activity and above all the substrate specificity of the enzyme. With regard to substitutions at the aromatic ring, the active‐site cavity appears to provide more space for attachment at C‐4 than at C‐5. This is in good agreement with the observed preference for substituents at C‐4 of the resorcinol core structure over those at the C‐5 position.16b

## Conclusion

The presented structural characterization of the multicomponent acytransferase *Pp*ATase reveals a close interaction of all three enzyme subunits PhlA, PhlB, and PhlC. However, the C−C bond formation without the utilization of CoA is performed through the action only of the PhlC subunit. This structural information provides a basis for developing libraries of catalysts tailored for specific chemical substrates. Our goal is to extend the substrate scope by using established protein engineering techniques.

## Experimental Section

**Expression and purification**: The CoA‐independent acyltransferase from *Pseudomonas protegens* DSM 19095 (*Pp*ATase) was expressed in *E. coli* as described previously16b from a plasmid containing codon‐optimized open reading frames (ORFs) coding for the three subunits of the enzyme: PhlA, PhlB, and PhlC. The nucleotide sequence of the expression plasmid is available from GenBank through the accession number KY173355. Purification of the enzyme was achieved by size‐exclusion chromatography as described previously.16b

**Crystallization and soaking**: Screening for crystallization conditions was performed with an Oryx8 crystallization robot (Douglas Instruments). Initial trials were set up by employing the sitting‐drop vapor‐diffusion method in 96‐well plates with Index HT (Hampton Research), JCSG+, and Morpheus (Molecular Dimensions) screens. A stock solution of *Pp*ATase [12 mg mL^−1^ in potassium phosphate buffer (pH 7.5, 50 mm)] was used for all crystallization experiments. Both in initial screens and in subsequent optimizations, drops (1 μL) were set with a 1:1 ratio of protein and precipitant solution. The crystallization plates were incubated at 289 K.

Crystal clusters of *Pp*ATase were readily obtained under several sets of conditions. Microseed‐matrix‐screening experiments^33^ were set up with initial crystals obtained under conditions Index #2 and #81 as seeding stock (1:1000 dilution, 0.1 μL added to the drop). With this technique, single crystals were obtained under conditions Index #91 [dl‐malic acid (pH 7.0, 0.15 m), poly(ethylene glycol) (3350, 20 %, *w*/*v*)] and #55 [magnesium chloride hexahydrate (0.1 m, 0.05 m), HEPES (pH 7.5), poly(ethylene glycol) monomethyl ether (550, 30 %, *v*/*v*)] after about one week. Soaking experiments were performed by adding small amounts of pure MAPG dissolved in DMSO directly to the crystallization drop with a cryoloop. Soaking times varied between 20 and 60 s. Untreated and soaked crystals were flash‐cooled in liquid nitrogen with use of glycerol (15 %, *v*/*v*) as cryoprotectant. Numerous crystals obtained under different crystallization conditions were tested for diffraction.

**Data collection, processing, structure determination, and analysis**: Data were collected at 100 K on synchrotron beamlines ID23‐1 and ID30B (ESRF, Grenoble, France)^34^ from an untreated crystal (hexagonal, space group *P*6_1_22) and from a crystal soaked with MAPG (orthorhombic, space group *P*2_1_2_1_2_1_), to crystallographic resolutions of approximately 2.8 and 3.4 Å, respectively. Diffraction data were processed and scaled by using the XDS package.^35^ Initial automated molecular replacement attempts with Balbes^36^ and the better resolved hexagonal dataset indicated the dimer of 3‐hydroxy‐3‐methylglutaryl‐coenzyme A synthase from *Staphylococcus aureus* (PDB ID: 1TVZ, 23 % sequence identity)^37^ as a suitable search template for PhlA. A homology model of PhlC was generated with the aid of the Phyre2 server^19^ with the structure of SCP2 thiolase from *Leishmania mexicana* (PDB ID: 3ZBG, 27 % sequence identity)^29^ as template. Molecular replacement was continued within the CCP4 suite^38^ by using the program Phaser.^39^ By fixing the previously positioned 1TVZ dimer (template for PhlA), two copies of the PhlC homology model could be placed in the asymmetric unit. Density modification based on phases from this partial model by using the program Resolve^40^ yielded well‐defined electron density for the whole *Pp*ATase complex, including density for two missing PhlB molecules. Manual rebuilding of PhlA and PhlC was continued with the aid of the program Coot,^41^ and the improved model was then subjected to automated rebuilding with the program Buccaneer.^42^ The resulting model containing two copies of each of PhlA, PhlB, and PhlC was completed manually in Coot and refined by using the PHENIX software suite.^43^

The structure of the *Pp*ATase soaked with MAPG was solved by molecular replacement with a part of the previously determined hexagonal structure (one copy of each of PhlA, PhlB, and PhlC) as search template. Structure solution resulted in eight copies of this trimeric arrangement in the asymmetric unit of the orthorhombic unit cell. Structure refinement was continued in the same manner as described above, with the programs Coot and PHENIX. Clear difference electron density was observed in all eight chains of PhlC in the vicinity of residue Cys88. In four of those chains we interpreted this density as a molecule of phloroglucinol. Additional density around Cys88 was interpreted as an acetyl group covalently attached to the Sγ atom of this amino acid residue.

For both structures, validation was performed with the program MolProbity.^44^ Data collection and refinement statistics are summarized in Table 2.

**Table 2:** Crystal structure of *Pp*ATase, data collection, and refinement statistics.

	*Pp*ATase	*Pp*ATase
	hexagonal^[a]^	orthorhombic
		(soaked)^[a]^
wavelength	0.8726	0.95
resolution range	48.74–2.83 (2.94–2.83)	49.72–3.44 (3.56–3.44)
space group	*P*6_1_22	*P*2_1_2_1_2_1_
Unit cell dimensions		
*a*, *b*, *c* [Å]	222.63, 222.63, 237.10	104.79, 229.78, 311.13
*α*, *β*, *γ* [°]	90, 90, 120	90, 90, 90
total reflections	69 5209 (67 477)	60 2513 (51 680)
unique reflections	82 036 (7914)	99 724 (9200)
multiplicity	8.5 (8.5)	6.0 (5.6)
completeness [%]	1.00 (0.98)	0.99 (0.93)
mean *I*/*σ*(*I*)	7.87 (2.23)	8.10 (2.57)
Wilson *B* factor	36.41	53.96
*R*_merge_	0.255 (0.891)	0.268 (0.788)
*R*_meas_	0.271 (0.948)	0.294 (0.868)
CC_1/2_	0.98 (0.67)	0.97 (0.81)
CC*	0.99 (0.90)	0.99 (0.95)
reflections used in	82 033 (7914)	99 695 (9198)
refinement		
reflections used for *R*_free_	4102 (395)	4986 (460)
*R*_work_	0.163 (0.251)	0.166 (0.219)
*R*_free_	0.204 (0.316)	0.219 (0.292)
CC_work_	0.96 (0.83)	0.95 (0.90)
CC_free_	0.94 (0.74)	0.91 (0.82)
number of non‐H atoms	14 084	54 563
macromolecules	13 594	54 398
ligands	20	116
protein residues	1800	7196
RMS (bonds)	0.011	0.005
RMS (angles)	0.91	0.72
Ramachandran favored [%]	96	97
Ramachandran allowed [%]	3.7	3.1
Ramachandran outliers [%]	0.11	0.18
rotamer outliers [%]	3.2	1.5
Clashscore	4.29	10.12
average *B* factor	21.32	43.64
macromolecules	21.24	43.63
ligands	25.53	52.78
solvent	23.51	30.28
PDB ID	5M3K	5MG5

[a] Statistics for the highest‐resolution shell are shown in parentheses.

All structure‐related figures were generated by using PyMOL (http://www.pymol.org). Structures were superimposed with the SSM Superposition tool^22^ as implemented in Coot. Cavities were identified in the final structures by using the LIGSITE algorithm^45^ as implemented in the CaSoX plugin for PyMOL. The analysis of the hydrophobicity of these cavities utilized the corresponding function of the program VASCo.^46^

**Site‐directed mutagenesis and activity measurements**: Variants of *Pp*ATase were prepared in order to investigate the roles of selected amino acid residues in the enzymatic reaction. Gene mutations were introduced with the aid of the QuickChange II site‐directed mutagenesis kit (Agilent Genomics) according to the standard procedure provided by the supplier without modifications. All primer sequences and plasmids used in this study are collated in Table S1. The following variants, each carrying a single amino acid exchange within *phlC*, were generated: H56A, H56S, N87A, C88A, C88S, H144A, H144S, W211A, W211F, Y298A, Y298V, Y298F, H347F, S349A, and D352V. All variants were expressed as described for the wild‐type enzyme16b and were used as cell‐free extracts in the activity measurements (Table 1).

The activities of wild‐type *Pp*ATase and of its variants were tested with the acetylation of benzene‐1,3‐diol (resorcinol) under the following conditions: Resorcinol (10 mm final concentration in the reaction mixture) was dissolved in potassium phosphate buffer (pH 7.5, 50 mm) and preheated to 35 °C for 10 min. Cell‐free extracts of the recombinant enzymes (30 mg protein) were subsequently added to the preheated mixture. The bioacetylation was started by addition of imidazole (100 mm, added from a 1 m stock solution prepared in the reaction buffer, causing the pH to increase to 8.30), followed by the addition of isopropenyl acetate (IPEA, 100 mm). To ensure proper suspension of the donor in the mixture, the vessel was manually shaken thoroughly right after starting the reaction. The reaction mixture (1 mL total volume) was horizontally shaken for 1.5 h at 35 °C and 750 rpm in an orbital shaker. Reactions were terminated by addition of acetonitrile (1 mL). The precipitated protein was removed by centrifugation (18 407 *g*, 10 min), and the supernatant (900 μL) was transferred to an Eppendorf tube and left standing for another 40 min. Any residual precipitated protein was once again removed by centrifugation, and the supernatant was directly subjected to HPLC for determination of degree of conversion. The relative amounts of resorcinol, the C‐acetylation product 4‐acetylresorcinol, and the O‐acetylation product 2(3‐hydroxyphenyl)acetate were determined by HPLC from standard curves with authentic samples. Each reaction was performed as a duplicate.

Specific activities were measured with a Thermo Scientific Genesys 10 UV Scanning UV/Vis spectrophotometer according to a modified procedure from the literature.13a When the disproportionation of MAPG into DAPG and PG is followed spectrophotometrically, an increase of absorption due to the formation of DAPG (*ϵ*=20 mm ^−1^ cm^−1^, *λ*=370 nm) is recorded. One unit of activity was defined as 1 μmol of product formed by an enzyme in 1 min per 1 milligram of protein under the following conditions: potassium phosphate buffer (pH 7.5, 100 mm, 960 μL) and MAPG (1.2 μmol, 30 μL of a 40 mm stock solution prepared in DMSO) were added to a cuvette and preheated to 35 °C. The reaction (1 mL total volume, 3 vol% DMSO) was started by the addition of the enzyme‐containing cell‐free extract (10 μL). The reaction was followed for 1 minute. All reactions were performed in duplicate. The protein concentration was measured with Bradford reagent (*ϵ*=0.083 mL mg^−1^ cm^−1^, *λ*=595 nm), and specific activities were determined as units per mg protein.

## Conflict of interest

*The authors declare no conflict of interest*.

## Supporting information

As a service to our authors and readers, this journal provides supporting information supplied by the authors. Such materials are peer reviewed and may be re‐organized for online delivery, but are not copy‐edited or typeset. Technical support issues arising from supporting information (other than missing files) should be addressed to the authors.

Supplementary
