# Supplementary material for: Structure and Catalytic Mechanism of a Bacterial Friedel–Crafts Acylase
Source: Chembiochem. 2018 Nov 26;20(1):88–95. doi: 10.1002/cbic.201800462 (PMC6392133; doi:10.1002/cbic.201800462)
Supplement: Supplementary file 1 — Supplementary [file CBIC-20-88-s001.pdf]

CHEMBIOCHEM

Supporting Information

Structure and Catalytic Mechanism of a Bacterial Friedel–Crafts Acylase

Tea Pavkov-Keller,[a, b] Nina G. Schmidt,[a, c] Anna Żądło-Dobrowolska,[c] Wolfgang Kroutil,*[a, c, d] and Karl Gruber*[a, b, d]

cbic_201800462_sm_miscellaneous_information.pdf

Author Contributions

T.P. Conceptualization: Equal; Data curation: Lead; Formal analysis: Lead; Funding acquisition: Equal; Investigation: Equal; Project administration: Equal; Visualization: Lead; Writing – original draft: Equal; Writing – review & editing: Equal

N.S. Data curation: Equal; Formal analysis: Equal; Investigation: Supporting; Writing – original draft: Supporting; Writing – review & editing: Supporting

A.Ż. Data curation: Equal; Formal analysis: Equal; Funding acquisition: Equal; Investigation: Supporting; Writing – original draft: Equal; Writing – review & editing: Equal

W.K. Conceptualization: Equal; Data curation: Supporting; Formal analysis: Supporting; Funding acquisition: Equal; Investigation: Equal; Project administration: Equal; Supervision: Equal; Writing – original draft: Equal; Writing – review & editing: Equal

K.G. Conceptualization: Equal; Data curation: Supporting; Formal analysis: Supporting; Funding acquisition: Equal; Investigation: Equal; Project administration: Equal; Supervision: Equal; Visualization: Supporting; Writing – original draft: Equal; Writing – review & editing: Equal.

Supporting Information Table 1. Plasmids and primers used in this study. Mutagenized codons are shown in bold, restriction sites are underlined. Ribosomal binding sites are shown in lowercase letters.

**Plasmids**	**Origin (GenBankID)**	**Description/Comments**
pASKIBA3plus	IBA-Lifescience	P_{Tet}, Amp^r, $ColE1_{ori}$, C-terminal StrepTag
pEG332	Schmidt *et al.*, 2017[1]	Codon-optimized gene fragments *phlA, phlC* and *phlB* based on *phlACB* from *P. protegens* DSM19095, assembled by Gibson cloning and overlap-extension PCR. PCR primers: OE1-4ATaseCH-Fow/Rev.
pEG332-C88A	this study	Single mutation C88A introduced to pEG332. PCR primers: *Pp*-C88A-Fow/Rev
pEG332-C88S	this study	Single mutation C88S introduced to pEG332. PCR primers: *Pp*-C88S-Fow/Rev
pEG332-C83A	this study	Mutation C83A introduced to pEG332. PCR primers: *Pp*-C83A-Fow/Rev
pEG332-H347F	this study	Mutation H347F introduced to pEG332. PCR primers: *Pp*-H347F-Fow/Rev
pEG332-D352V	this study	Mutation D352V introduced to pEG332. PCR primers: *Pp*-D352V-Fow/Rev
pEG332-S349A	this study	Mutation S349A introduced to pEG332. PCR primers: *Pp*-S349A-Fow/Rev
pEG332-F148A	this study	Mutation F148A introduced to pEG332. PCR primers: *Pp*-F148A-Fow/Rev
pEG332-F148V	this study	Mutation F148V introduced to pEG332. PCR primers: *Pp*-F148V-Fow/Rev
pEG332-L383A	this study	Mutation L383A introduced to pEG332. PCR primers: *Pp*-L383A-Fow/Rev
pEG332- L383V	this study	Mutation L383V introduced to pEG332. PCR primers: *Pp*-L383V-Fow/Rev
pEG332-Y298A	this study	Mutation Y298A introduced to pEG332. PCR primers: *Pp*-Y298A-Fow/Rev
pEG332- Y298V	this study	Mutation Y298V introduced to pEG332. PCR primers: *Pp*-Y298V-Fow/Rev
pEG332- Y298F	this study	Mutation Y298F introduced to pEG332. PCR primers: *Pp*-Y298F-Fow/Rev
pEG332-N87A	this study	Mutation N87A introduced to pEG332. PCR primers: *Pp*-N87A-Fow/Rev
pEG332-W211A	this study	Mutation W211A introduced to pEG332. PCR primers: *Pp*-W211A-Fow/Rev
pEG332-W211F	this study	Mutation W211F introduced to pEG332. PCR primers: *Pp*-W211F-Fow/Rev
pEG332-H144A	this study	Mutation H144A introduced to pEG332. PCR primers: *Pp*-H144A-Fow/Rev
pEG332- H144S	this study	Mutation H144S introduced to pEG332. PCR primers: *Pp*- H144S-Fow/Rev
pEG332- H56A	this study	Mutation H56A introduced to pEG332. PCR primers: *Pp*-H56A-Fow/Rev
pEG332- H56S	this study	Mutation H56S introduced to pEG332. PCR primers: *Pp*-H56S-Fow/Rev

**Primers**	**Origin**	**Sequence (5'→3')**
*Pp*WT-Fow	Eurofins	ATATAGGTACCATGAACGTGAAAAAGATAGGTATTG
*Pp*WT-Rev	Eurofins	ATATAGGATCCTTATATATCGAGTACGAACTTATAAG
*Pb*WT-Fow	Eurofins	ATATAGGTACCATGAATAAAGTAGGAATTGTG
*Pb*WT-Rev	Eurofins	ATATAGGATCCTTATTTCACCAGTACAAACTTATAG
OE1ATaseCH-Fow	IDT	ATATAAGAATTCaaggagatatacataTGATGAATGTGAAGAAAATAGGTATCGTTAGC
OE2ATaseCH-Fow	IDT	CGCTGACCGCGTACCTCTAAGGTACCaaggagatatacataTGATGTGCGCACGTCGCG
OE3ATaseCH -Rev	IDT	TGCGCACATCAtatgtatatctccttGGTACCTTAGAGGTACGCGGTCAGCGCATAATC
OE4ATaseCH -Rev	IDT	ATATATGAATTCGCCGAGACGGCCATG
Sequencing-Fow	Eurofins	TTGTTCTGTGCGGCGGTTTT
Sequencing-Rev	Eurofins	CTTAGATCTCGTCGACTGAGT
Sequencing-1158-Fow	Eurofins	AAGCCAGGAAGTTCACGAGT
Sequencing-pASK-IBA3plus-Fow	Eurofins	GAGTTATTTTACCACTCCCT
Sequencing-pASK-IBA3plus-Rev	Eurofins	CGCAGTAGCGGTAAACG
Pp-C83A-Fow	IDT	GCTGTATCTGGGCACC**GCC**ACCAACCCATACGAT
Pp-C83A-Rev	IDT	ATCGTATGGGTTGGT**GGC**GGTGCCCAGATACAG
Pp-H347F-Fow	IDT	TATTGGCCGCGGC**TTC**GCGTCTGGCTGC
Pp-H347F-Rev	IDT	GCAGCCAGACGC**GAA**GCCGCGGCCAATA
Pp-D352V-Fow	IDT	CGCGTCTGGCTGC**GTT**GGCATTCTGCATA
Pp-D352V-Rev	IDT	TATGCAGAATGCC**AAC**GCAGCCAGACGCG

Pp-S349A-Fow	IDT	GCGGCCACGCG**GCT**GGCTGCGAT
Pp-S349A-Rev	IDT	ATCGCAGCC**AGC**CGCGTGGCCGC
Pp-F148A-Fow	Eurofins	CTCATACCGACGCG**GC**TGCGCTGGCGACCG
Pp-F148A-Rev	Eurofins	CGGTCGCCAGCGCA**GC**CGCGTCGGTATGAG
Pp-F148V-Fow	Eurofins	TCATACCGACGCG**G**TTGCGCTGGCGAC
Pp-F148A-Rev	Eurofins	GTCGCCAGCGCAA**C**CGCGTCGGTATGA
Pp-L383A-Fow	Eurofins	CGCATTGGCGTGTCGCAGAAT**GC**TGGCGGTTATG
Pp-L383A-Rev	Eurofins	CATAACCGCCA**GC**ATTCTGCGACACGCCAATGCG
Pp-L383V-Fow	Eurofins	CATTGGCGTGTCGCAGAAT**G**TTGGCGGTTATGC
Pp-L383V-Rev	Eurofins	GCATAACCGCCAA**C**ATTCTGCGACACGCCAATG
Pp-Y298A-Fow	Eurofins	ATTGATGTGGCGCAGGTT**GC**TGATCTGCTCGGCGC
Pp-Y298A-Rev	Eurofins	GCGCCGAGCAGATCA**GC**AACCTGCGCCACATCAAT
Pp-Y298V-Fow	Eurofins	ATTGATGTGGCGCAGGTT**GT**TGATCTGCTCGGCGC
Pp-Y298V-Rev	Eurofins	GCGCCGAGCAGATCA**AC**AACCTGCGCCACATCAAT
Pp-Y298F-Fow	Eurofins	GATGTGGCGCAGGTTT**T**TGATCTGCTCGGC
Pp-Y298F-Rev	Eurofins	GCCGAGCAGATCA**A**AAACCTGCGCCACATC
Pp-N87A-Fow	Eurofins	CTTTATGAGCACCGCG**GC**TTGCACCAGCAGCTCG
Pp-N87A-Rev	Eurofins	CGAGCTGCTGGTGCAA**GC**CGCGGTGCTCATAAAG
Pp-W211A-Fow	Eurofins	CTCGATGCTGGCG**GC**GGGCGAAGCGAGT
Pp-W211A-Rev	Eurofins	ACTCGCTTCGCCC**GC**CGCCAGCATCGAG
Pp-W211F-Fow	Eurofins	GGCTCGATGCTGGCGT**TC**GGCGAAGCGAG
Pp-W211F-Rev	Eurofins	CTCGCTTCGCC**GA**ACGCCAGCATCGAGCC
Pp-H144A-Fow	Eurofins	CTTTCTCGGCATCTCT**GC**TACCGACGCGTTTGCG
Pp-H144A-Rev	Eurofins	CGCAAACGCGTCGGTA**GC**AGAGATGCCGAGAAAG
Pp-H144S-Fow	Eurofins	ACTTTCTCGGCATCTCT**AG**TACCGACGCGTTTGCGC
Pp-H144S-Rev	Eurofins	GCGCAAACGCGTCGGTA**CT**AGAGATGCCGAGAAAGT
Pp-H56A-Fow	Eurofins	GGTGGCGTACGGTTAC**GC**TGGTGAAGGCATCTCG
Pp-H56A-Rev	Eurofins	CGAGATGCCTTCACCA**GC**GTAACCGTACGCCACC
Pp-H56S-Fow	Eurofins	GGTGGCGTACGGTTAC**AG**TGGTGAAGGCATCTCG
Pp-H56S-Rev	Eurofins	CGAGATGCCTTCACCA**CT**GTAACCGTACGCCACC

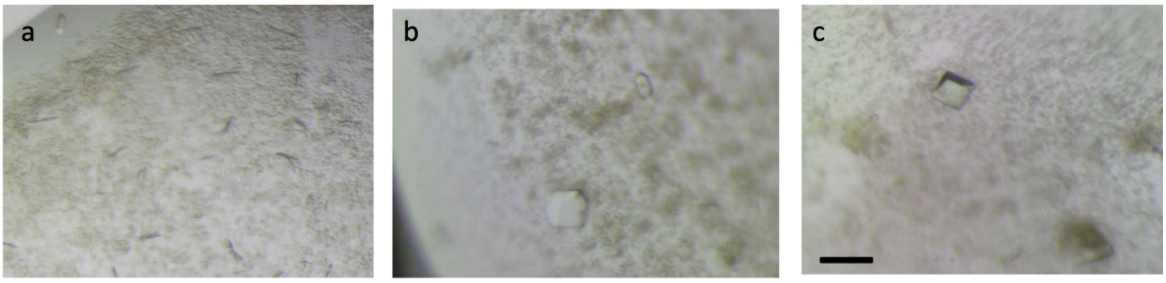

Supporting Information Figure 1. Crystals of *Pp*ATase. **(a)** Initial crystals, used for microseed matrix-screening experiments. (**b**, **c)** Two different crystal forms. Native data were collected on crystal B ($P6_122$) and soaking was performed with crystal C ($P2_12_12_1$); bar=100 µm.

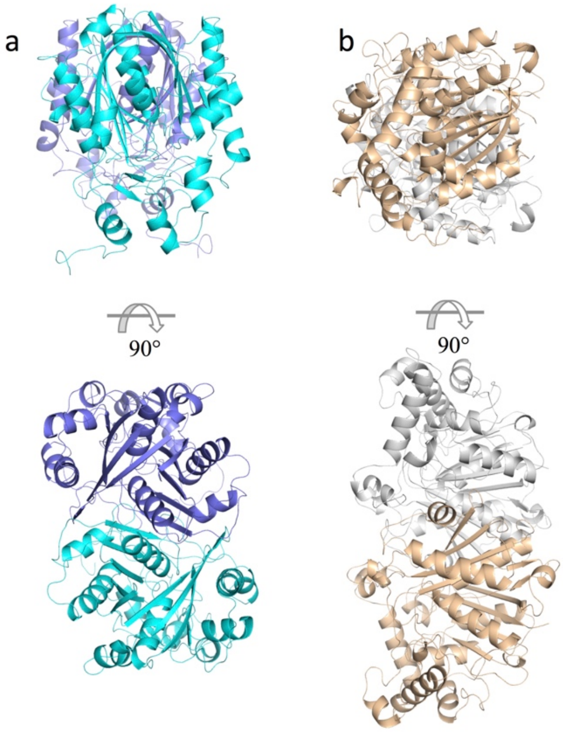

Supporting Information Figure 2. Structural analysis of *Pp*ATase. (**a**) Dimers of PhlA (blue, cyan) and (b) PhlC (grey, wheat) as present in the dodecameric arrangement observed in both crystal structures. Individual chains are shown in cartoon representation.

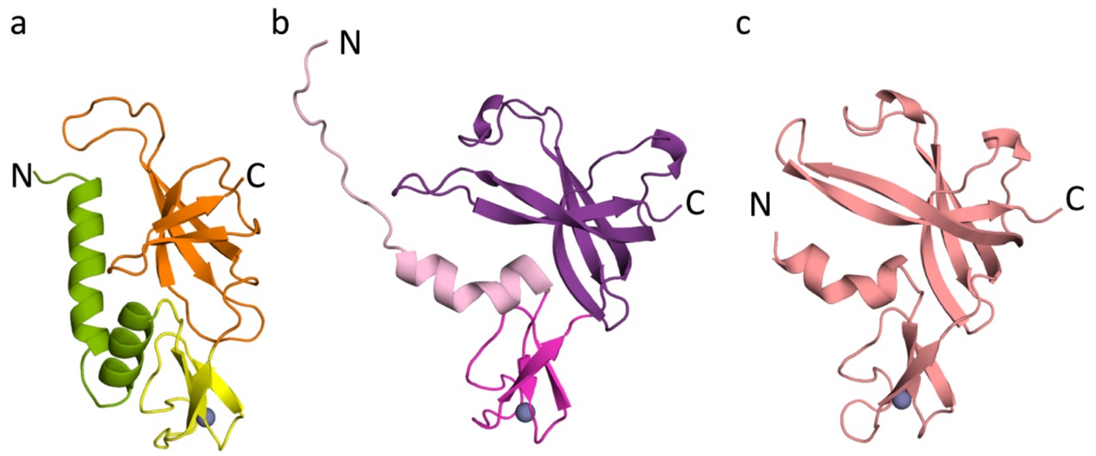

Supporting Information Figure 3. Structure comparison of the (a) protein from DUF35 family from *Sulfolobus solfataricus* (PDB: 3irb, seq id: 18%)[2] and (b) PhlB. Proteins show a two-domain architecture comprising a N-terminal helical fragment (light pink and green), rubredoxin-like zinc ribbon (magenta and yellow) and a C-terminal oligonucleotide/oligosaccharide-binding (OB) fold domain (violet and orange). The structure of (c) DUF35 family protein from *Methanothermococcus thermolithotrophicus* (PDB: 6et9, seq id: 26.7 %)[3] is shown in salmon. Zink atom is shown in violet sphere.

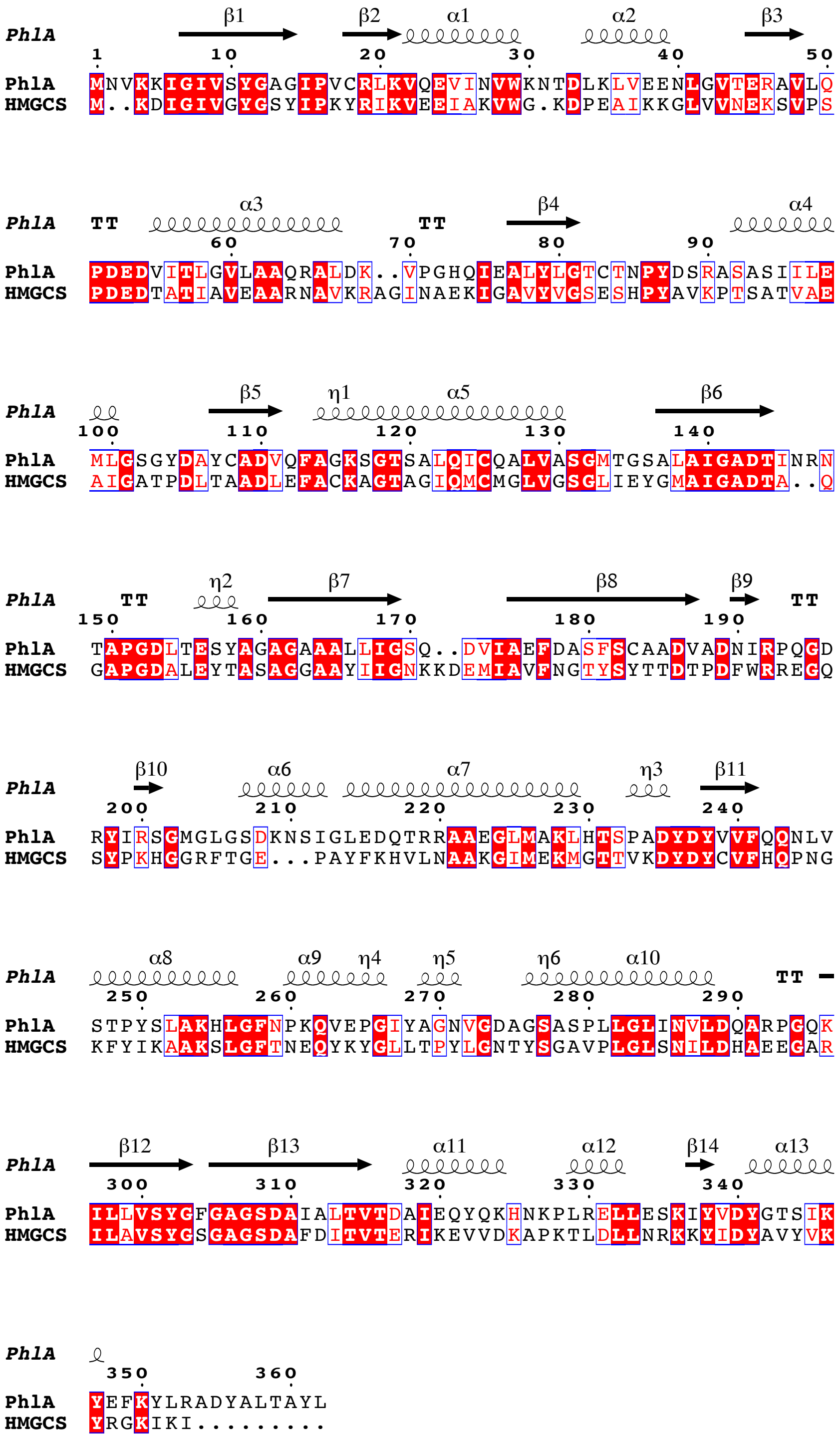

Supporting Information Figure 4: Sequence alignment of the PhlA subunit of *Pp*ATase and the HMGCS domain of the acetoacetyl-CoA thiolase/HMG-CoA synthase from *Methanothermococcus thermolithotrophicus*.[3] The alignment was generated using the program T-Coffee[4] and displayed using the ENDscript server[5].

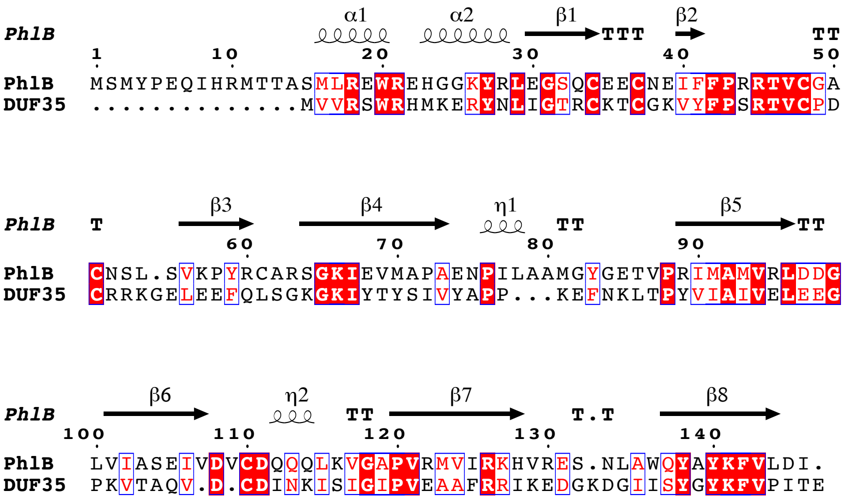

Supporting Information Figure 5: Sequence alignment of the PhlB subunit of *Pp*ATase and the DUF35-family type subunit of the acetoacetyl-CoA thiolase/HMG-CoA synthase from *Methanothermococcus thermolithotrophicus*.[3] The alignment was generated using the program T-Coffee[4] and displayed using the ENDscript server[5].

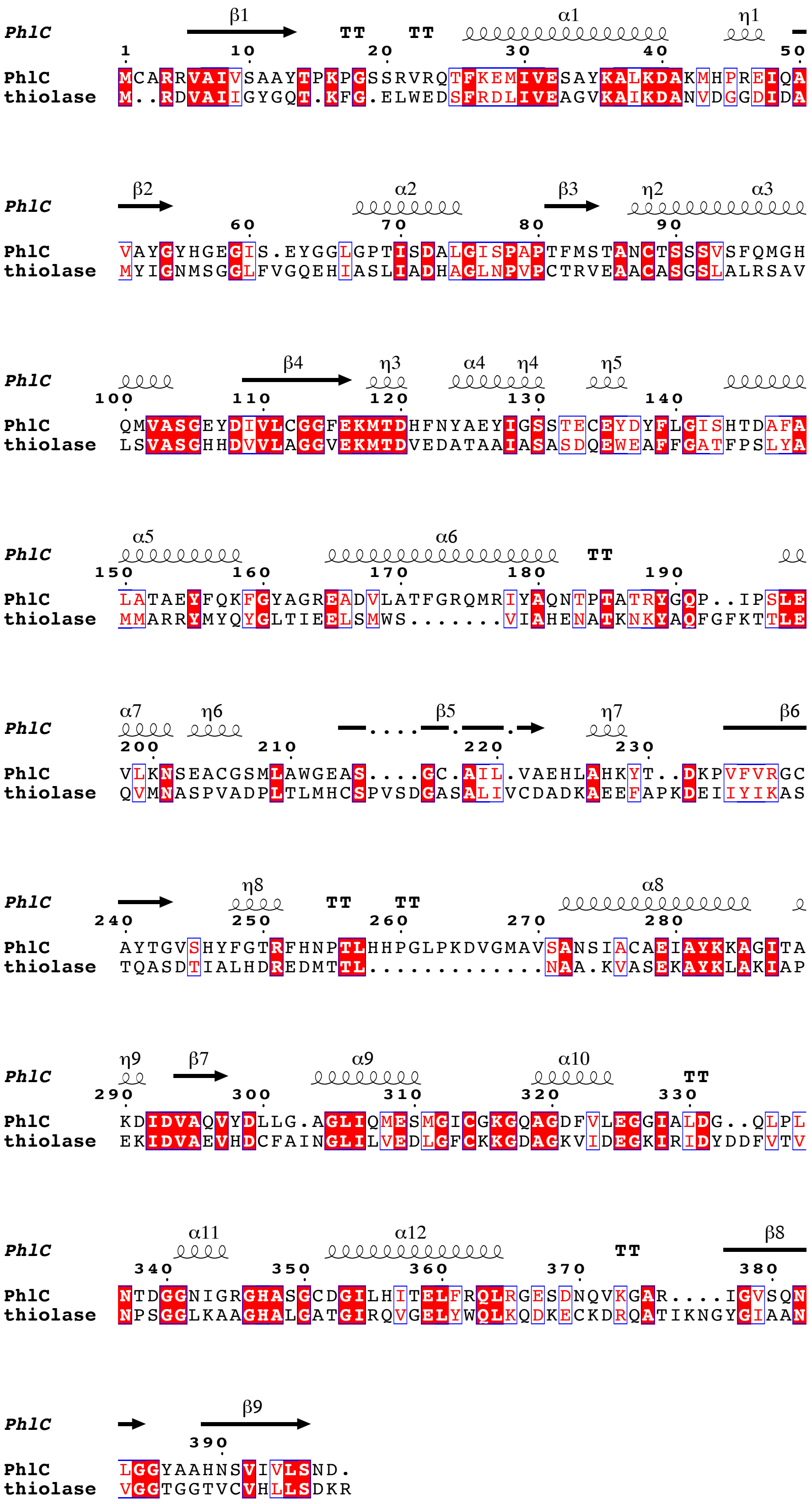

Supporting Information Figure 6: Sequence alignment of the PhlC subunit of *Pp*ATase and the thiolase subunit of the acetoacetyl-CoA thiolase/HMG-CoA synthase from

Methanothermococcus thermolithotrophicus.[3] The alignment was generated using the program T-Coffee[4] and displayed using the ENDscript server[5].

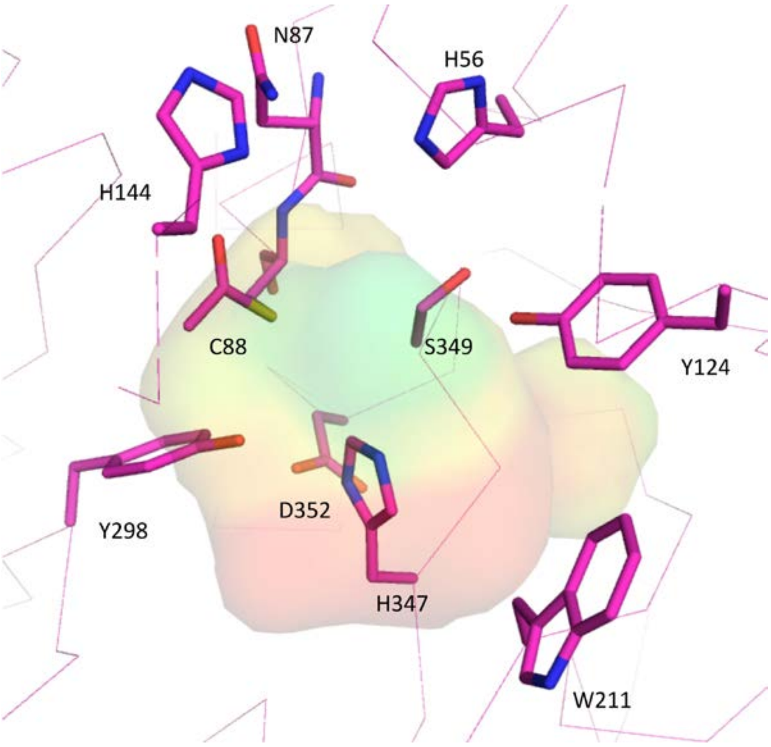

Supporting Information Figure 7. Amino acid residues in the PhlC active site subjected to mutagenesis studies are shown in a sticks representation. The active site cavity was calculated using Casox and is shown in a transparent surface presentation (red-hydrophobic to blue-hydrophilic).

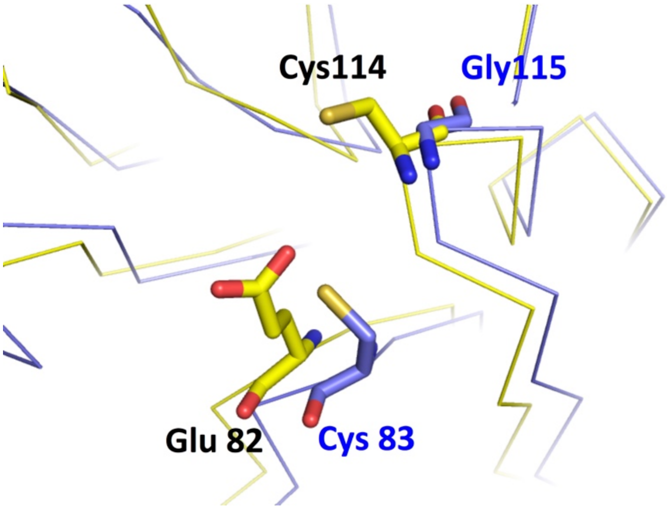

Supporting Information Figure 8. Comparison of PhlA (PpATase) with the HMGCS subunit of an archaeal acetoacetyl-CoA thiolase/HMG-CoA synthase complex from *Methanothermococcus thermolithotrophicus*, as present in PDB-entry 6esq. The catalytic residues (Cys114 and Glu82) important for HMGCS activity, *i.e.* the condensation of acetoacetyl-CoA and acetyl-CoA to 3-hydroxy-3-methylglutaryl-CoA, are shown in a sticks representation (yellow). The corresponding residues in PhlA (Gly115 and Glu82) are shown as blue sticks.

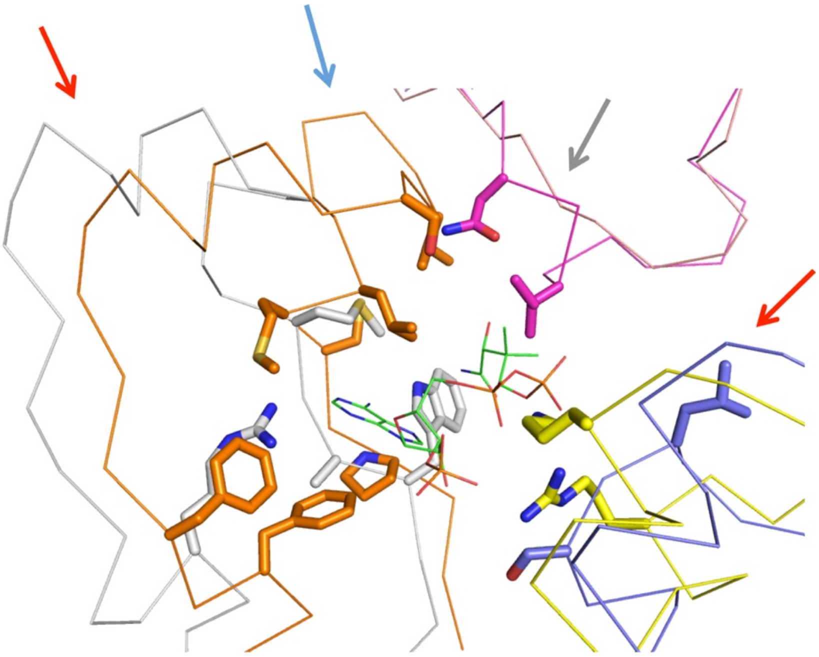

Supporting Information Figure 9. Comparison of *Pp*ATase with thiolase/HGSCM complex CoA binding region, as present in PDB 6esq (PhlA=blue, PhlB=magenta, PhlC=grey; thiolase=orange, HGSCM=yellow, DUF35 family protein= salmon; CoA=green). CoA is shown in green lines and residues within this region in stick representation. Red arrows – indicate positional differences; blue arrow – addition of two short beta strands in HGSCM; grey arrow – additional residues within PhlB.

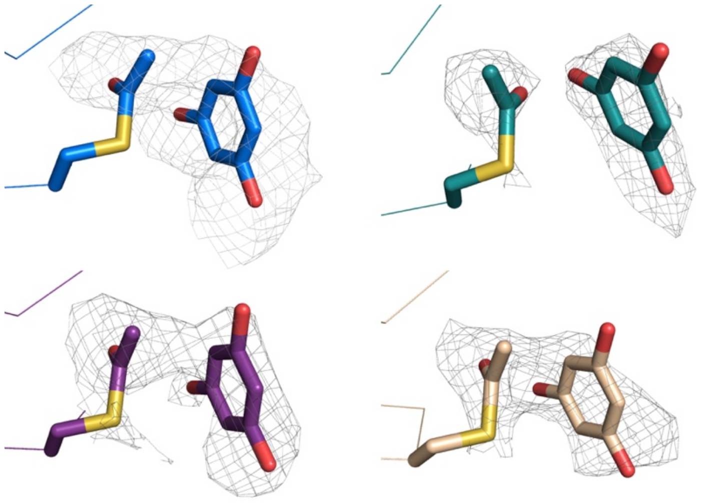

Supporting Information Figure 10. Electron density maps of the complex structure. Fo-Fc omit density is shown for four PhlC chains (contoured at 2σ). Amino acid residue Cys88 and the bound ligand are shown as sticks.

References

[1] N. G. Schmidt, T. Pavkov-Keller, N. Richter, B. Wiltschi, K. Gruber, W. Kroutil, *Angew. Chem. Int. Ed.* **2017**, *56*, 7615-7619.

[2] S. S. Krishna, L. Aravind, C. Bakolitsa, J. Caruthers, D. Carlton, M. D. Miller, P. Abdubek, T. Astakhova, H. L. Axelrod, H. J. Chiu, T. Clayton, M. C. Deller, L. A. Duan, J. Feuerhelm, J. C. Grant, G. W. Han, L. Jaroszewski, K. K. Jin, H. E. Klock, M. W. Knuth, A. Kumar, D. Marciano, D. McMullan, A. T. Morse, E. Nigoghossian, L. Okach, R. Reyes, C. L. Rife, H. van den Bedem, D. Weekes, Q. P. Xu, K. O. Hodgson, J. Wooley, M. A. Elsliger, A. M. Deacon, A. Godzik, S. A. Lesley, I. A. Wilson, *Acta Cryst. F* **2010**, *66*, 1160-1166.

[3] B. Vögeli, S. Engilberge, E. Girard, F. Riobé, O. Maury, T. J. Erb, S. Shima, T. Wagner, *Proc. Natl. Acad. Sci. USA* **2018**, *115*, 3380-3385.

[4] C. Notredame, D. G. Higgins, J. Heringa, *J. Mol. Biol.* **2000**, *302*, 205-217.

[5] X. Robert, P. Gouet, *Nucleic Acids Res.* **2014**, *42*, W320-W324.
